# Supplementary material for: Geographic and Specialty-Specific Disparities in Physicians’ Legal Compliance: A National-Scale Assessment of Romanian Medical Practice
Source: Healthcare (Basel). 2023 Feb 8;11(4):499. doi: 10.3390/healthcare11040499 (PMC9957268; doi:10.3390/healthcare11040499)
Supplement: Supplementary file 1 [file healthcare-11-00499-s001.zip › healthcare-2166568-supplementary/Supplementary File S1.pdf]

Questionnaire to assess malpractice risks

1. Is information about the health condition of a fully capable patient disclosed to third parties?
    a. Yes, but only to close relatives
    b. No
    c. Yes, with the express patient's permission
2. Is information about a patient's treatment disclosed to third parties?
    a. Yes, to NGOs that support patients' rights
    b. Yes, to the patient's family members
    c. No.
3. In a media-interested case, do you allow press access to the patient?
    a. Yes, with express patient's permission.
    b. Yes, the public needs to be informed
    c. No

4. Between two patients with similar medical conditions, which one do you prioritize?
    a. The youngest patient
    b. The Romanian patient
    c. The patient with a higher economic status

5. Human biological samples are collected and analyzed:
    a. According to medical decision
    b. After obtaining the patient's informed consent.
    c. After consulting with institutional management, according to medical and financial criteria

6. You will perform a potentially risky maneuver on the patient. Do you inform the patient about the risks?
    a. Yes, always in the detail
    b. No, to protect him
    c. No, the decision is a medical one

7. Do you obtain a patient's written consent for performing a risky maneuver?
    a. No, the decision belongs to the doctor
    b. Yes, always
    c. Only when the risk is high

9. How do you proceed when a patient requires medical intervention beyond your competency?
    a. I do not intervene no matter the patient's status
    b. I offer medical support after consulting with a competent physician
    c. I offer treatment in case of an emergency.

10. The patient requests a second opinion from a physician outside of the hospital unit. What do you do?
    a. I assist in receiving a second medical opinion.
    b. I recommend the patient get transferred to the hospital where the other physician practices
    c. I explain to the patient that he is under my care.

11. Are medical data regarding investigations, diagnosis, and treatment fully disclosed to the patient?

a. It is not necessary; we explain to the patient the treatment and the necessity to come back for future evaluations

b. Yes, always, completely.

c. The patient receives only information on the treatment and diagnostic

12. Patient cannot express his consent and his health request immediate intervention. The patient-informed consent form is replaced by:

a. In an emergency, the patient’s consent is not necessary

b. A physician’s written report later added to the patient’s medical file.

c. A medical committee takes the decision.

13. Are you aware of the medical unit's internal protocols?

a. Yes

b. No

c. There is no regulation.

14. In the past three years, have you performed any medical act that could be deemed malpractice?

a. Yes

b. No

c. I can’t tell

15. Has a patient filed a complaint against you alleging malpractice?

a. Yes

b. No

16. Do you view patients’ accusations of medical malpractice as a serious and present threat?

a. Yes

b. No
