# Supplementary material for: Geographic and Specialty-Specific Disparities in Physicians’ Legal Compliance: A National-Scale Assessment of Romanian Medical Practice
Source: Healthcare (Basel). 2023 Feb 8;11(4):499. doi: 10.3390/healthcare11040499 (PMC9957268; doi:10.3390/healthcare11040499)
Supplement: Supplementary file 1 [file healthcare-11-00499-s001.zip › healthcare-2166568-supplementary/Supplementary File S2.pdf]

Appendix B – Detailed results

Results General Practitioners

Region	Total	1		2		3		4		5		6		7		9		10		11		12	
		No	%	No	%	No	%	No	%	No	%	No	%	No	%	No	%	No	%	No	%	No	%
Bucuresti If	180	81	45,00	78	43,33	63	35,00	129	71,67	39	21,67	173	96,11	77	42,78	77	42,78	136	75,56	85	47,22	35	19,44
Centru	164	71	43,29	55	33,54	93	56,71	124	75,61	34	20,73	157	95,73	61	37,20	85	51,83	124	75,61	57	34,76	24	14,63
NE	182	63	34,62	47	25,82	100	54,95	120	65,93	42	23,08	152	83,52	127	69,78	39	21,43	111	60,99	53	29,12	36	19,78
NV	192	82	42,71	71	36,98	83	43,23	133	69,27	34	17,71	182	94,79	75	39,06	92	47,92	153	79,69	79	41,15	28	14,58
Sud	296	107	36,15	111	37,50	127	42,91	204	68,92	45	15,20	280	94,59	138	46,62	124	41,89	223	75,34	91	30,74	61	20,61
SE	216	109	50,46	93	43,06	110	50,93	145	67,13	56	25,93	206	95,37	95	43,98	102	47,22	150	69,44	73	33,80	36	16,67
SV	162	87	53,70	78	48,15	75	46,30	96	59,26	23	14,20	151	93,21	65	40,12	78	48,15	117	72,22	44	27,16	28	17,28
Vest	168	86	51,19	64	38,10	95	56,55	115	68,45	27	16,07	165	98,21	70	41,67	84	50,00	121	72,02	61	36,31	42	25,00
TOTAL	1560	686	43,97	597	38,27	746	47,82	1066	68,33	300	43,73	1466	93,97	708	45,38	681	43,65	1135	72,76	543	34,81	290	18,59

Self-assessment

Region	1							2							3					4			
	Yes	%	No	%	There is none/I cannot appreciate	%	Total	Da	%	No	%	There is none/I cannot appreciate	%	Total	Yes	%	No	%	Total	Yes	%	No	%
Bucuresti If	108	64,29	26	15,48	34	20,24	168	13	7,83	74	44,58	79	47,59	166	14	7,53	172	92,47	186	162	87,10	24	12,90
Centru	116	70,73	32	19,51	16	9,76	164	14	8,43	75	45,18	77	46,39	166	9	5,49	155	94,51	164	158	96,34	6	3,66
NE	114	65,52	29	16,67	31	17,82	174	18	10,78	71	42,51	78	46,71	167	34	19,54	140	80,46	174	162	93,10	12	6,90
NV	131	65,83	41	20,60	27	13,57	199	18	8,82	74	36,27	112	54,90	204	12	5,88	192	94,12	204	192	94,58	11	5,42
Sud	181	61,15	73	24,66	42	14,19	296	23	7,77	126	42,57	147	49,66	296	21	6,60	297	93,40	318	301	94,95	16	5,05
SE	119	55,35	71	33,02	25	11,63	215	27	12,56	79	36,74	109	50,70	215	24	11,32	188	88,68	212	207	96,28	8	3,72
SV	78	56,93	28	20,44	31	22,63	137	15	9,38	78	48,75	67	41,88	160	17	10,63	143	89,38	160	148	92,50	12	7,50
Vest	115	68,45	33	19,64	20	11,90	168	7	4,17	80	47,62	81	48,21	168	7	4,17	161	95,83	168	161	95,83	7	4,17
TOTAL	962	63,25	333	21,89	226	14,86	1521	135	8,75	657	42,61	750	48,64	1542	138	8,70	1448	91,30	1586	1491	93,95	96	6,05

Attending Physicians

Region	Total	1		2		3		4		5		6		7		9		10		11		12	
		No	%	No	%	No	%	No	%	No	%	No	%	No	%	No	%	No	%	No	%	No	%
Bucuresti If	305	97	31,80	67	21,97	122	40,00	181	59,34	57	18,69	286	93,77	251	82,30	60	19,67	206	67,54	109	35,74	70	22,95
Centru	130	40	30,77	24	18,46	64	49,23	93	71,54	22	16,92	129	99,23	105	80,77	29	22,31	83	63,85	46	35,38	28	21,54
NE	188	61	32,45	37	19,68	105	55,85	114	60,64	40	21,28	179	95,21	151	80,32	21	11,17	124	65,96	62	32,98	36	19,15
NV	178	55	30,90	32	17,98	72	40,45	97	54,49	53	29,78	172	96,63	151	84,83	41	23,03	116	65,17	62	34,83	37	20,79
Sud	207	58	28,02	31	14,98	98	47,34	121	58,45	45	21,74	194	93,72	158	76,33	56	27,05	126	60,87	68	32,85	49	23,67
SE	165	47	28,48	29	17,58	98	59,39	92	55,76	48	29,09	159	96,36	139	84,24	36	21,82	100	60,61	48	29,09	43	26,06
SV	74	21	28,38	18	24,32	35	47,30	46	62,16	13	17,57	72	97,30	61	82,43	17	22,97	38	51,35	26	35,14	19	25,68
Vest	144	37	25,69	23	15,97	74	51,39	90	62,50	29	20,14	139	96,53	129	89,58	30	20,83	93	64,58	53	36,81	39	27,08
TOTAL	1391	416	29,91	261	18,76	668	48,02	834	59,96	307	73,80	1330	95,61	1145	82,31	290	20,85	886	63,70	474	34,08	321	23,08

Self-assessment

Region	1							2							3					4			
	Yes		No		There is none/I cannot appreciate		Total	Yes		No		There is none/I cannot appreciate		Total	Yes		No		Total	Yes		No	
Bucuresti If	151	52,80	81	28,32	54	18,88	286	37	12,67	125	42,81	130	44,52	292	69	22,70	235	77,30	304	292	96,37	11	3,63
Centru	81	64,80	18	14,40	26	20,80	125	22	17,74	50	40,32	52	41,94	124	24	18,60	105	81,40	129	121	93,80	8	6,20
NE	103	66,03	31	19,87	22	14,10	156	22	14,10	75	48,08	59	37,82	156	46	28,40	116	71,60	162	155	95,68	7	4,32
NV	130	62,50	48	23,08	30	14,42	208	23	10,90	100	47,39	88	41,71	211	52	24,41	161	75,59	213	192	89,30	23	10,70
Sud	120	60,91	48	24,37	29	14,72	197	31	15,35	97	48,02	74	36,63	202	76	37,81	125	62,19	201	193	94,15	12	5,85
SE	99	55,62	48	26,97	31	17,42	178	24	13,56	71	40,11	82	46,33	177	58	32,40	121	67,60	179	168	92,82	13	7,18
SV	48	55,81	22	25,58	16	18,60	86	8	9,20	42	48,28	37	42,53	87	32	34,78	60	65,22	92	86	93,48	6	6,52
Vest	93	58,86	31	19,62	34	21,52	158	27	16,27	83	50,00	56	33,73	166	41	24,40	127	75,60	168	161	96,41	6	3,59
TOTAL	825	59,18	327	23,46	242	17,36	1394	194	13,71	643	45,44	578	40,85	1415	398	27,49	1050	72,51	1448	1368	94,09	86	5,91
